# An Evolutionary Conserved Signaling Network Between Mouse and Human Underlies the Differential Osteoskeletal Potential of Frontal and Parietal Calvarial Bones

**DOI:** 10.3389/fphys.2021.747091

**Published:** 2021-10-21

**Authors:** Siddharth Menon, Julika Huber, Chris Duldulao, Michael T. Longaker, Natalina Quarto

**Affiliations:** ^1^Hagey Laboratory for Pediatric Regenerative Medicine, Department of Surgery, School of Medicine, Stanford University, Stanford, CA, United States; ^2^Division of Plastic and Reconstructive Surgery, Department of Surgery, Stanford University School of Medicine, Stanford, CA, United States; ^3^Institute for Stem Cell Biology and Regenerative Medicine, Stanford University School of Medicine, Stanford, CA, United States; ^4^Department of Plastic Surgery, University Hospital Bergmannsheil Bochum, Bochum, Germany; ^5^Dipartimento di Scienze Biomediche Avanzate, Università degli Studi di Napoli Federico II, Napoli, Italy

**Keywords:** evolution, osteoskeletal, signaling, calvarial, bones

## Abstract

The mammalian calvarial vault is an ancient and highly conserved structure among species, however, the mechanisms governing osteogenesis of the calvarial vault and how they might be conserved across mammalian species remain unclear. The aim of this study was to determine if regional differences in osteogenic potential of the calvarial vault, first described in mice, extend to humans. We derived human frontal and parietal osteoblasts from fetal calvarial tissue, demonstrating enhanced osteogenic potential both *in vitro* and *in vivo* of human frontal derived osteoblasts compared to parietal derived osteoblasts. Furthermore, we found shared differential signaling patterns in the canonical WNT, TGF-β, BMP, and FGF pathways previously described in the mouse to govern these regional differences in osteogenic potential. Taken together, our findings unveil evolutionary conserved similarities both at functional and molecular level between the mouse and human calvarial bones, providing further support that studies employing mouse models, are suitable for translational studies to human.

## Introduction

The vertebrate skull comprises the neuro-cranium, the viscero-cranium, the sclerotomal occipital region and the dermal skull roof. The neuro-cranium is composed of the skull base comprising cartilaginous parts, sensory capsules and the central part of the skull roof. The viscero-cranium (or splanchnocranium), the pharyngeal arch skeletons forming the face, composes also the jaw, supporting the feeding structures (Morriss-Kay, 2001). Both, the neuro-cranium and viscero-cranium contain cartilaginous elements comprising the chondrocranium.

The vertebrate dermal skull roof is an ancient structure, protecting the brain and extending laterally to the sides. Description of the skull roof can be found as early as in species like agnathan fossil fishes, a species remnant of a primitive offshoot of the vertebrates (Caputo Barucchi et al., 2013). The skull roof includes the intramembranous paired-frontal, paired-parietal and post-parietal bones, adding the squamosal bone, a part of the sphenoid and the supraoccipital bone, this early tetrapod skull evolved to a mammalian skull. As the brain grows, these cover bones expand by way of the fibrous sutures connecting the bones (Morriss-Kay, 2001; Quarto and Longaker, 2005).

The anatomy of the mammalian skull is highly conserved across several species, such as the human and murine (Opperman, 2000; Quarto and Longaker, 2005), and molecular biology and microanatomy investigations have provided evidence that the correlation and putative developmental link between brain-regions and bony-skull elements are likely to be ancestral to Tetrapoda (Fabbri et al., 2017).

It is well established that the skull roof is partly of mesodermal origin, and substantially of neural crest origin. Earlier studies in avian followed later by studies in mice have defined the dual embryonic tissue origins of the skull bones (Le Lièvre and Le Douarin, 1975; Jiang et al., 2002; Yoshida et al., 2008). More recent studies from our group have unveiled the significant impact of this dual embryonic tissue origin on the osteo-skeletal potential of the neural crest-derived frontal bone and mesoderm-derived parietal bone (Quarto et al., 2009, 2010; Li et al., 2010, 2013). By investigating these regional embryonically determined differences, we have previously identified distinct/differential domains of endogenously active pro-osteogenic signaling as governing substantial differences between the osteoskeletal potential of murine frontal and parietal bones and derived osteoblasts. Specifically, signaling such as FGF, BMP, and canonical Wnt (cWnt) were highly activated in both, murine frontal bones and derived osteoblasts (FOb) as compared to parietal bones and derived osteoblasts (POb) (Quarto et al., 2009, 2010; Li et al., 2010, 2013). Conversely, TGF-β signaling was the signaling solely activated at higher extent in parietal bones and POb (Li et al., 2013).

On the bases of these previous findings, herein moving a step forward, we addressed whether differences in osteoskeletal profiles of murine FOb and POb represent a conserved osteoskeletal signature. Our current study unveiled that like murine FOb and POb, human osteoblasts derived from frontal and parietal bones display substantial differences in their osteogenic activity, as well a differential activation of the pro-osteogenic FGF, BMP, cWNT, and TGF-β-signaling pathways.

## Results

### Human Frontal and Parietal Bones-Derived Osteoblasts Have Different Osteogenic Potential *in vitro*

**FIGURE 1:**
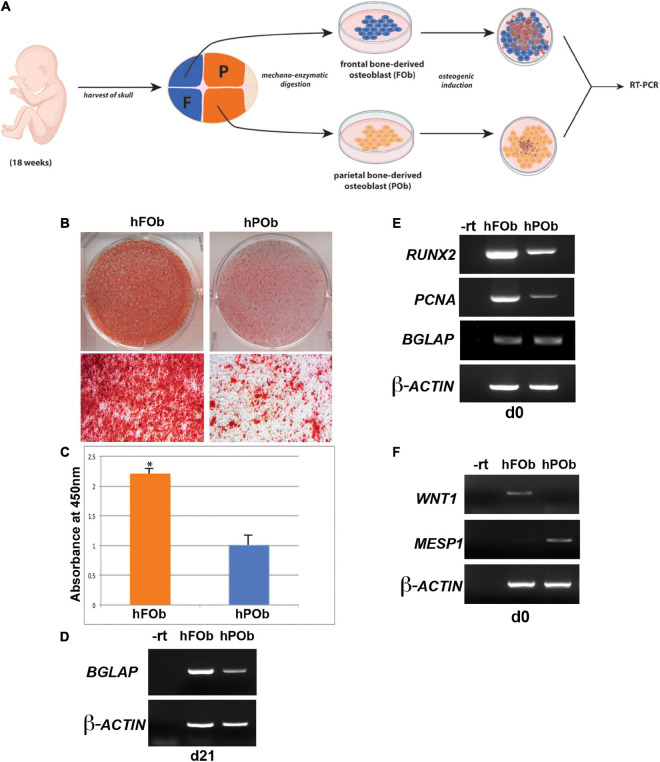
Differential osteogenic potential is hallmark of hFOb and POb. **(A)** Schematic representation of the experimental procedures. Abbreviations: F, Frontal bone; P, Parietal bone. **(B)** *In vitro* osteogenic differentiation of hFOb and hPOb prepared from fetal (18-weeks) human frontal and parietal bone. Alizarin red staining performed at day 21 of the osteo-induction assay showed a more robust mineralization of the extracellular matrix (ECM) in hFOb as compared to hPOb. **(C)** Quantification of Alizarin red staining further confirmed a more abundant mineralization of the ECM in hFOb, *p-value < 0.05 **(D)** RT-PCR expression analysis of the late marker of osteogenesis, *BGLAP* (*OSTEOCALCIN*) revealed higher expression of this gene in hFOb then hPOb (at day 21), thus suggesting a more advanced terminal osteogenic differentiation of hFOb relatively to hPOb. **(E)** RT-PCR expression analysis of the osteoprogenitors marker *RUNX2* at day 0, prior osteo-induction assay showed a major increase in the expression of this gene in hFOb than hPOb, thus indicating the presence of a larger pool of osteoprogenitors in hFOb. Elevated levels of *PCNA* paralleled the increased expression of *RUNX2* in hFOb as well, suggesting a higher proliferative activity of hFOb as compared to hPOb. Conversely, no differences in the expression levels of *BGLAP* were observed between the two types of osteoblasts. **(F)** RT-PCR expression analysis showing unique expression of the neural crest marker *WNT1* in hFOb but not in hPOb, whereas expression of the mesoderm marker *MESP1* is observed only in hPOb.

Our previous work unveiling distinct differences between the osteoskeletal ability of the neural crest-derived mouse calvarial frontal bone and mesoderm-derived parietal bone, as well derived osteoblasts, prompted us to investigate whether these differences are evolutionarily conserved in the same human calvarial bones as well. To address this question, we first analyzed the osteogenic activity of human fetal derived frontal bone osteoblasts (hFOb) and parietal bone derived osteoblasts (hPOb) *in vitro*. Primary cell cultures of osteoblasts freed from pericranium and dura mater cells were prepared from the frontal and parietal calvarial bones of human fetal specimens (18-weeks gestation, approximately). An *in vitro* osteogenic assay was performed to assess the osteogenic potential of hFOb and hPOb (Figure 1A). After 21 days, the extent of osteo-induction and extracellular matrix mineralization was evaluated by alizarin red staining. As shown in Figure 1B, increased mineralization and larger bone nodule formation were observed in hFOb as compared to hPOb. The more robust mineralization of hFOb was also confirmed by quantification of alizarin red staining (Figure 1C) reading higher absorbance at 450nm. RT-PCR analysis for the expression of the late osteogenic marker *BGLAP* (*Osteocalcin*) at day 21 revealed higher expression in hFOb relatively to hPOb, thus, further confirming the greater terminal osteogenic differentiation achieved by hFOb in comparison to hPOb (Figure 1D). Remarkable, as previously observed in murine frontal bone–derived osteoblasts, hFOb also expressed higher levels of *RUNX2*, an early marker of osteogenic differentiation and osteoprogenitors, in addition to increased expression of *Proliferating Cell Nuclear Antigen* (*PCNA*) (Figure 1E). This observation suggested the presence of a larger pool of osteoprogenitor cells with higher proliferative activity in hFOb as compared to hPOb. In contrast, both hFOb and hPOb expressed equal levels of *BGLAP* at time day 0, prior *in vitro* osteo-induction, suggesting an equal representation of a mature/late osteoblasts pool. Collectively, these results unveiled an *in vitro* osteoskeletal profile of hFOb and hPOb mirroring that described by our earlier studies in mouse (Quarto et al., 2009, 2010; Li et al., 2010, 2013). Of note, RT-PCR analysis of the neural crest marker *WNT1* and mesodermal marker *MESP1* revealed a uniquely differential expression between hFOb and hPOb (Figure 1F). These two markers have been previously employed for lineage tracing defining the dual neural crest and mesoderm origin of calvarial bones in transgenic mice (Jiang et al., 2002; Yoshida et al., 2008).

### Enhanced *in vivo* Bone Regeneration of Critical Size Mouse Calvarial Defects by Transplanted Human Frontal Bone Derived Osteoblasts

**FIGURE 2:**
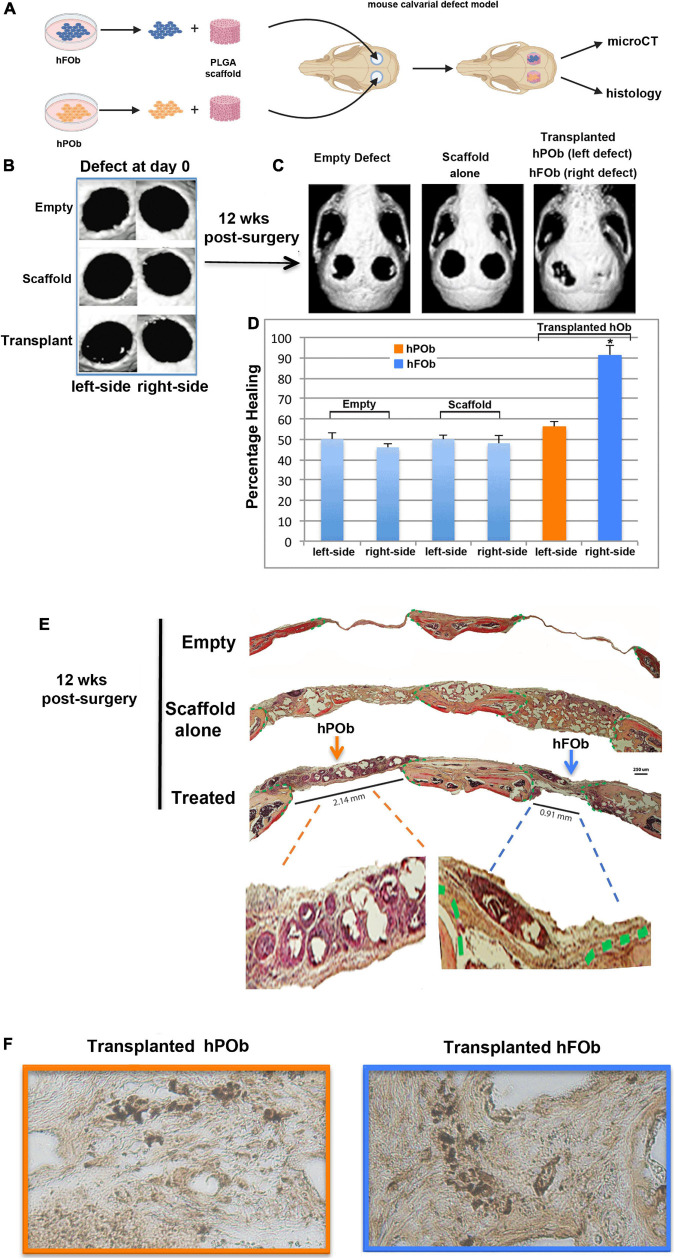
*In vivo* transplanted hFOb and hPOb feature different osteoskeletal regenerative ability. **(A)** Cartoon depicting the scheme of the experimental procedures. **(B)** Representative images of micro-CT (μCT) acquired at day 0 (24 h upon defects creation Four-millimeter calvarial defects were created on both sides of parietal bone in 9-weeks old CD-1 nude mice (*n* = 5/each group). Defects were treated with a PLGA scaffold loaded either with hFOb (right side) or hPOb (left side), number of transplanted cells was 2.5 × 10^5^. Animal groups with empty defect or PLGA scaffold alone were implemented as control to assess the extent of endogenous bony tissue repair. **(C)** Skull defects regeneration images obtained by μCT analysis at 12-weeks post-surgery. **(D)** Quantification of bone regeneration determined by μCT at 12-weeks post-surgery. For statistical analysis, empty group and scaffold alone group was compared with other groups using the Mann-Whitney *U*-test. A *p-value < 0.05 was considered statistically significant. **(E)** Pentachrome staining of coronal sections showing healing in parietal defects treated with transplanted hFOb, neo-formed bony-tissue (yellow-color) bridging the calvarial defect edges can be appreciated. Conversely defects treated with POb did not promote any bony tissues formation. Green dashed lines mark the osteogenic fronts surrounding the defects. Pentachrome stains bony tissue in yellow. Scale bar values 250 μm. **(F)** Human nuclear antigen staining detecting the presence of both hFOb and hPOb within the area of calvarial defects creation at 12-weeeks post-surgery.

Next, we evaluated the regenerative capacity of transplanted hFOb and hPOb *in vivo*. To this end, two critical size (4 mm) calvarial defects were created on each side of the parietal bones of 9-week-old CD-1 nude mice (Figure 2A). The choice to create a calvarial defect on parietal bone rather than frontal was suggested by the diminished regenerative ability of the murine parietal bone critical size defect compared to the frontal bone critical size defect (Quarto et al., 2010). Therefore, based on this knowledge, we reasoned that a wounded parietal bone would provide a more optimal background to evaluate intrinsic regenerative activity of transplanted hFOb and hPOb *in vivo*. Figure 2B shows representative images of micro-CT (μCT) acquired 24 h upon defects creation (referred as d0). Passage-1 osteoblasts were seeded on a PGLA scaffold and transplanted into the defect, hFOb into the right-side defect and hPOb into the left-side defect, respectively. Empty defects and scaffold alone were used as negative controls. Bone formation was evaluated at 12-weeks post-surgery by micro-CT (μCT) and histology. As illustrated in Figure 2C **(Left panel)**, both, left and right empty defects did not heal as expected with a critical size defect (Aalami et al., 2004). Defects treated with scaffold alone also did not heal Figure 2C **(Middle panel**). In contrast, defects treated with hFOb healed to near completion, while those treated with hPOb did not (Figure 2C, **Right panel**). Percentage of healing is illustrated by the histogram (Figure 2D). These results were further supported by histological analysis of coronal sections encompassing both right and left defects. Movat’s pentachrome staining revealed regeneration of bony tissue (yellow color) in the defects treated only with hFOb as compared to hPOb and control animal groups (Figure 2E). Transplanted FOb supported bone bridging at the interphase between the edges of the bone defect. Human nuclear antigen staining detected both hFOb and hPOb transplanted in the defects area at 12-weeks post-surgery, thus demonstrating the survival of both cell types (Figure 2F), however, only hFOb promoted bone repair. Collectively, these data suggested that hFOb have higher intrinsic osteoskeletal regenerative potential than hPOb.

### Human and Murine Frontal Bones Derived Osteoblasts and Parietal Bones Derived Osteoblasts Share a Similar Differential Activation Profile of Key Pro-osteogenic Signaling

Extensive investigation has previously identified the integration of differential activation of several pro-osteogenic signaling pathways as a major player in governing the regional differences in the osteoskeletal potential between mouse FOb and POb (Quarto et al., 2009, 2010; Li et al., 2010, 2013). Having identified similar differences for the *in vitro* osteogenic activity between hFOb and POb we next interrogated whether these human osteoblasts share similarity with murine FOb and POb in terms of specific endogenous activate pro-osteogenic signaling.

**FIGURE 3:**
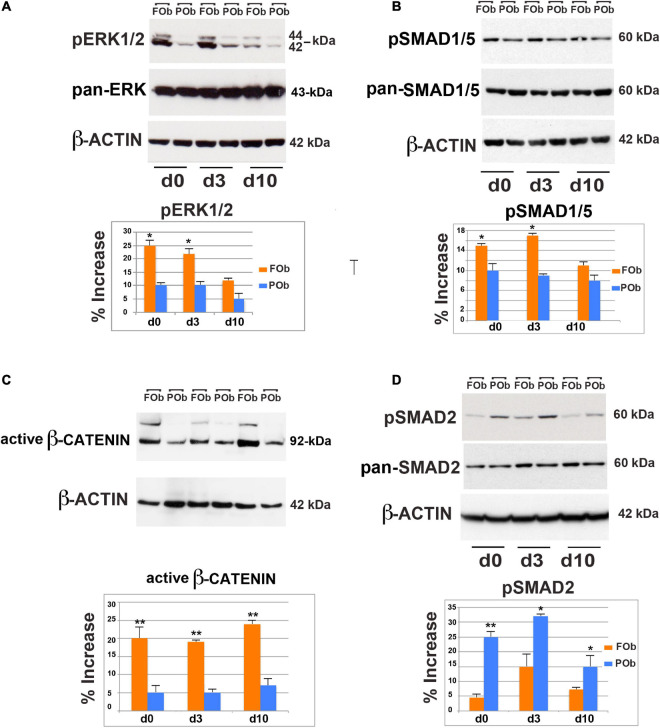
Integration of multiple pro-osteogenic signaling pathways defines distinct active hFOb and hPOb. **(A)** Time-course immuno-detection of phospho-ERK1/2 (pERK1/2) in hFOb and hPOb undergoing to osteogenic differentiation showing enhanced active pERK overtime in hFOb in comparison to hPOb. Pan-ERK and β-ACTIN antibody probing the same membrane were used as internal controls for equal loading and transfer of the samples. **(Bottom panel)**, histogram represents the densitometric analysis of electrophoresis bands using the Image J program; the relative intensities of bands were normalized to their respective loading control and set as 100%. **(B)** Time-course immuno-detection of phospho-SMAD1/5 (pSMAD1/5) proteins in hFOb and hPOb undergoing osteogenic differentiation showing increased protein levels in hFOb than hPOb. Immunoblotting with pan-SMAD1/5 and β-ACTIN antibody were employed as controls for equal loading and transfer of each sample. **(Bottom panel)**, histogram represents the densitometric analysis of electrophoresis bands using the Image J program; the relative intensities of bands were normalized to their respective loading control and set as 100%. **(C)** Time-course immuno-detection of increased active β-catenin protein in hFOb is indicative of an enhanced activation of cWNT signaling in hFOb as compared to hPOb. **(D)** Time-course immuno-detection of phospho-SMAD2/3 (pSMAD2/3) in hFOb and hPOb undergoing osteogenic differentiation shows on the contrary of what observed for the above signaling analyses, enhanced activation of TGF-β signaling as assessed by high levels of pSMAD2/3 in hPOb then hFOb. **(Bottom panel)**, Immuno-detection of pan-SMAD2/3 and β-ACTIN was used as internal controls as described above **(A,B)**. **(Bottom panel)**, histogram represents the densitometric analysis of electrophoresis bands using the Image J program; the relative intensities of bands were normalized to their respective loading control and set as 100%. Values: *p ≤ 0.05, **p ≤ 0.01.

We first, investigated the extent of FGF-mediated signaling in hFOb and hPOb during osteogenic differentiation on d0, d3, and d10 by immunoblotting analysis of phospho-ERK (pERK), a downstream effector for several signaling, included the FGF pathway signaling (Chaudhary and Avioli, 1997; Quarto et al., 2009; Li et al., 2010). This analysis revealed higher protein levels of pERK1/2 in hFOb vs. hPOb at d0, d3, and d10 of osteogenic differentiation, however, at d10 there was a decline of pERK in hFOb. Protein levels of pan-ERK were similar between hFOb and hPOb through the entire time course analysis thus ruling out that pERK differences observed between hFOb and hPOb could be a result of differences in pan-ERK protein levels (Figure 3A). These data demonstrated that hFOb cells, likewise mouse FOb, are endowed with higher levels of pERK1/2 proteins than POb.

BMP signaling is an additional key osteogenic-regulator that we found enhanced in murine FOb as compared to POb (Kanzler et al., 2000; Chen et al., 2004; Noël et al., 2004; Li et al., 2013). Therefore, we explored this signaling in human FOb and POb. As shown in Figure 3B, immunoblotting analysis detected increased levels of phospho-SMAD1/5 (pSMAD1/5) in hFOb vs. hPOb, although differences were less dramatic than what observed for pERK.

The cWnt pathway is a master regulator of differentiation and proliferation, regulating the fate determination of osteo/chrondro-progenitors (Logan and Nusse, 2004; Nusse, 2005, 2008; Hartmann, 2006; Krishnan et al., 2006; Davis and Zur Nieden, 2008; Monroe et al., 2012; Clevers et al., 2014). Enhanced activation of cWnt signaling in murine FOb further marks the greater osteoskeletal potential of murine FOb relatively to POb (Quarto et al., 2010). Therefore, we explored whether this signaling would also be enhanced in hFOb relatively to hPOb. To assess this, we performed an immunoblotting analysis for the active β-catenin using an antibody detecting the active form of β-catenin dephosphorylated on Ser37 or Thr41. A time course analysis during the osteogenic assay of hFOb and POb, showed an increase of active β-catenin in hFOb compared to hPOb, starting at time d0 and during the osteogenic assay d3 and d10 with a peak by day 10 (Figure 3C), thus mirroring murine FOb and POb profiles (Quarto et al., 2010).

Thus, a comparison of the extent of activation of the three pro-osteogenic signaling pathways FGF, BMP, and cWNT revealed a strong similarity between the mouse and human FOb and POb, with hFOb enhanced activation of all three signaling.

Interestingly, our previous investigations unveiled that TGF-β signaling was activated at larger extent in murine POb in comparison to FOb (Li et al., 2013). Remarkable, this distinct endogenous activation of TGF-β signaling was also confirmed in human osteoblasts. This is illustrated in Figure 3D showing data from an immunoblotting analysis probing for phospho-SMAD2/3 (pSMAD2/3) protein levels. Increased levels of endogenous pSMAD2/3 levels were already detected at d0 prior initiation of osteo-inductive assay, and levels remained sustained through the entire time course analysis.

Collectively, results stemming from the above analyses revealed a stringent similarity regarding the differentially activated signaling calvarial-domains between murine and human FOb and POb.

## Discussion

The conserved architecture of the mammalian calvarial vault raises several questions regarding the mechanisms governing osteogenesis of the human calvarial vault and how they might be conserved or different among mammalian species. Our previous work characterizing the osteoskeletal potential of the neural crest-derived frontal bone and mesoderm-derived parietal bone in mouse, established the increased osteogenic potential of the neural crest-derived frontal bone compared to the mesoderm-derived parietal bone (Quarto et al., 2010). This finding highlighted the importance of ontogeny of the frontal and parietal bones in determining their osteoskeletal potential and regenerative ability.

Herein, we hypothesized that regional differences in osteoskeletal potential observed in the mouse extends to the human fetal calvarial skull. To verify this hypothesis, we established primary osteoblast cultures from human fetal frontal (hFOb) and parietal bones (hPOb) and evaluated their osteoskeletal potential both, *in vitro* as well *in vivo* by transplantation into a calvarial defect created in mice. This investigation unveiled an enhanced osteoskeletal activity of hFOb in comparison to hPOb, thus mirroring studies in mice. *In vitro* osteo-inductive assays revealed robust mineralization of the extracellular matrix in hFOb as compared to POb and more advanced osteogenic terminal differentiation as assessed by expression of *BGLAP*. Furthermore, μCT data showed a nearly complete healing of calvarial defects transplanted with hFOb, a valuable finding highlighting the intrinsic osteoskeletal ability of hFOb at superior levels than hPOb.

Our previous studies in mice also indicated that enhanced activation of both FGF and Wnt/β-Catenin signaling pathways in frontal bones endows this tissue an enrichment of osteoprogenitors, and therefore higher osteogenic potential (Quarto et al., 2010). To evaluate if this might be true in human, we evaluated the expression level of *RUNX2*, an early marker of osteogenic differentiation and osteoprogenitors, as well a target of both FGF and Wnt/β-catenin signaling. Levels of *RUNX2*, and *PCNA* a proliferation marker, were both elevated in the hFOb compared to hPOb thus, suggesting an increased pool of osteoprogenitors.

**FIGURE 4:**
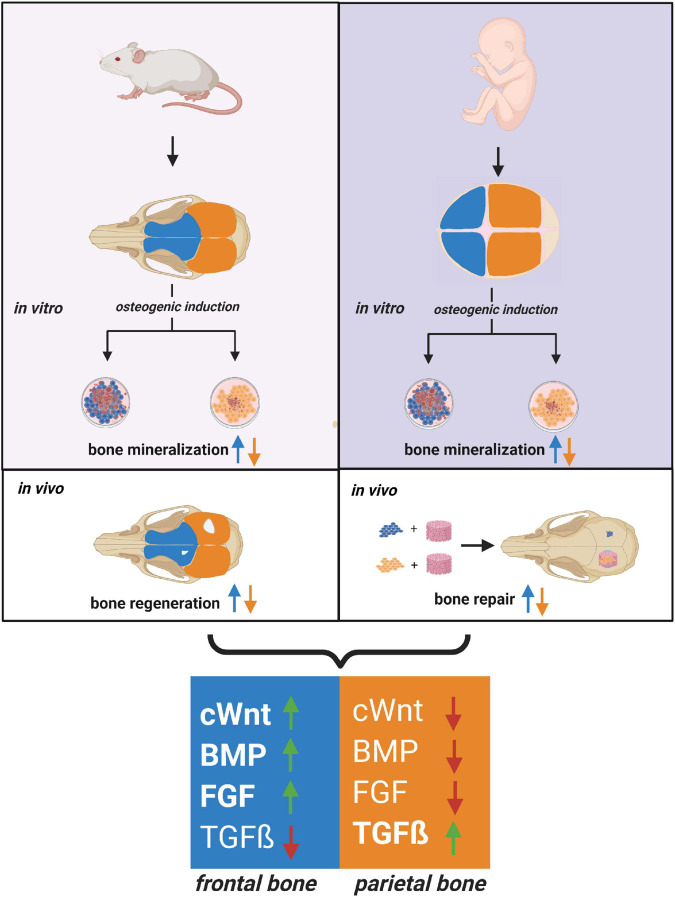
Relationship between human and mouse calvarial bones of neural crest and mesodermal origin. Cartoon depicting a close relationship between the human and mouse osteoskeletal capacity of frontal and parietal bones and –derived osteoblast. Similarities between the pro-osteogenic signaling pathways differentially activated in frontal and parietal bones-derived osteoblasts are schematically illustrated. Code colors: blue defines frontal bone and derived osteoblast; orange defines parietal bone and derived osteoblast.

The current study also mapped a distinct pattern of signaling pathways differentially activated in hFOb and hPOb resembling that previously described in mouse (Quarto et al., 2009, 2010; Li et al., 2010, 2013), and likewise, confirmed the integration of multiple differentially activated signaling. The latter finding unveiled a conserved signaling network acting as master regulator of a differential osteoskeletal potential in both, murine and human calvarial frontal and parietal bones (Figure 4).

The superior osteoskeletal activity of the murine neural crest-derived frontal bone compared to mesoderm-derived parietal bone can be traced as early embryonic stage 17.5 and it prolonged from early postnatally through adult stages (Quarto et al., 2009). This finding would strongly suggest that the higher osteoskeletal activity observed in hFOb compared to hPOb is a feature restricted not only to human fetal bones but toward the adult life-span.

The relevance of embryonic tissue origins in determining the regional difference of the murine frontal and parietal bones in relation to their osteoskeletal potential is well established (Quarto et al., 2009, 2010; Li et al., 2010, 2013) however, this has yet to be defined in human calvarial bones. Given the parallels found in osteogenic capacity and shared signaling patterns across mouse and human, it is reasonable to believe that human frontal bone is of neural crest origin and the parietal bone of mesoderm origin. This hypothesis also finds support from the mutually exclusive expression of the neural crest determinant *WNT1* in hFOb, and mesoderm determinant *MESP1* in hPOb (Jiang et al., 2002; Yoshida et al., 2008). However, this finding deserves further investigation. In this context, a noteworthy transcriptomic profiling study performed on two groups of human fetal cranial tissues: frontal and metopic compartments vs. parietal and sagittal compartments (Homayounfar et al., 2015), identified a gene expression signature of neural crest origin in frontal and metopic compartments while the gene expression signature of parietal and sagittal compartments shared more similarity to mesoderm (Homayounfar et al., 2015).

Furthermore, our findings also align with two previous studies showing that either an ecto-mesenchymal stem cell (eMSC) population-derived from human embryonic stem cells through a neural crest intermediate or induced human neural crest cell-mesenchymal progenitor cells (iNCC-MPCs) promoted new and enhanced bone formation in murine calvarial defects as compared to mesoderm-derived bone marrow mesenchymal stem cells (BM-MSCs) (Glaeser et al., 2021; Srinivasan et al., 2021). Taken all together, these findings strongly suggest the importance of taking in account the cell-origin when selecting a cell-source for bone regeneration applications.

Overall, the data stemming from our study revealed an evolutionary conserved similarity at functional and molecular level between mouse and human calvarial frontal and parietal bones. Considering the various limitations, both scientific and ethical, around the use of human post-natal and fetal tissue, our study provides further evidence that mammalian animal models, particularly the mouse, are valuable and suitable tools for translational studies to human in the context of bone homoeostasis and regeneration.

## Materials and Methods

### Human Frontal and Parietal Bones-Derived Osteoblasts Primary Culture

Human fetal skulls were purchased from Stemexpress (Folsom, CA) by overnight shipping. Samples ranged in age from 14 to 18 weeks of gestation with no restrictions on race or gender. Fetal sample procurement and handling was according to Stanford Institutional Review Board guidelines (IRB-35711). Under sterile conditions, periosteum and dura-mater tissues were stripped, skulls were then dissected under a stereomicroscope (Zeiss, Oberkochen, Germany) to obtain frontal and parietal bones. The dissected frontal and parietal bones were washed with dilute betadine and PBS and finely minced by a scissor prior to undergo to enzymatic digestion with 0.2% Dispase II and 0.1% Collagenase A (Roche Diagnostics, Indianapolis, IN, United States) in serum-free medium. The digestion was carried out six-times, each 15 min at 37°C in a water bath shaker. The first two digestions were discarded to avoid a potential contamination by residual pericranium and dura-mater derived cells. The last four digestions were pooled together upon neutralization with an equal volume of α-MEM supplemented with 10% fetal calf serum (FCS), 100 IU/ml penicillin and streptomycin (Gibco Life Technologies and Invitrogen Corporation, Carlsbad, CA), pelleted and resuspended in the growth medium as above. Human frontal and parietal bones-derived osteoblasts referred to as hFOb and hPOb were plated in 100 mm tissue culture dishes and incubated at 37°C with continuous supplement of 5% CO_2_. The growth medium was changed every 3 days. Osteoblasts were passaged by standard trypsinization procedure. Only passage 0 through 3 cells were employed for all experiments.

### *In vitro* Osteogenic Assay

For the *in vitro* osteo-induction assay hFOb and hPOb were plated in 6-well-plate (1 × 10^5^/well). Upon sub-confluence cells were incubated in the osteogenic differentiation medium (ODM), made of α-MEM supplemented with 10 μM glycerol β-phosphate, 0.25 μM ascorbic acid, (Sigma Aldrich, St. Louis, MO), 10% FCS, and 1% penicillin/streptomycin. Medium was changed every 2 days. At day 21 of the differentiation assay, Alizarin Red staining and its quantification was performed as previously described (Quarto et al., 2015) to assess the extent of extracellular matrix mineralization in hFOb and hPOb. hFOb and hPOb plates were air-dried and analyzed by light microscopy using an inverted microscope Leica DMI 4000B (Leica Biosystems Inc., Buffalo Grove, IL). Images were acquired using a ScanJet 5370C scanner (Hewlett-Packard Company, Palo Alto, CA).

### RT-PCR Analysis for Genes Expression

To analyze the expression level of specific genes RNAs were isolated from cells by Trizol procedure (Ambion-Life Technologies, Carlsbad, CA, United States) at different time points as indicated, and submitted to RT-PCR procedure as previously described (Quarto et al., 2015). Primers sequence and PCR conditions for *RUNX2, BGLAP*, and *ß-ACTIN* genes were previously described (Quarto et al., 2012; Senarath-Yapa et al., 2016). Additional primer sequences for other genes analyzed were designed based on their GenBank sequence. Primers sequences and annealing temperatures are listed in Table 1. Experiments were repeated two times.

**TABLE 1:** List of primers sequence and their annealing temperature.

**Gene**	**Accession number**	**Forward and reverse sequence**	**Size**	**Annealing temp**
*MESP1*	NM_018670.3	**Forward:** 5′-CTATATCGGCCACCTGTCGG-3′ **Reverse:** 5′-TCTGCCAAGGAACCACTTCG-3′	492 bp	58°C
*WNT1*	NM_005430.4	**Forward:** 5′- CCGCAACTATAAGAGGCGGT-3′ **Reverse:** 5′- ACGATCTTGCCGAAGAGGTG-3′	563 bp	58°C
*OSTERIX1 (SP7)*	NM_001173467.3	**Forward:** 5′- GAGTGGAACAGGAGTGGAGC-3′ **Reverse:** 5′- TGCCCCCATATCCACCACTA-3′	970 bp	58°C
*AXIN2*	NG_012142	**Forward:** 5′- GGTGCCCTACCATTGACACA-3′ **Reverse:** 5′- TGGGGATTCAAAGACGAGGC-3′	859 bp	58°C
*MYC*	NG_007161	**Forward:** 5′- TTGGCACGTCATATAGGCGA-3′ **Reverse:** 5′- GCCTGACTTTCGGGAAGGAA-3′	400 bp	57.5°C
*CCND1*	NG_007375	**Forward:** 5′- AGTGTGTCTTACGTGCCACC-3′ **Reverse:** 5′- TGCTCCTGGCTGGATTCTGT-3′	936 bp	58°C
*PCNA*	NG_047066	**Forward:** 5′- CAGTTCCCTTAGCAGCCCAG-3′ **Reverse:** 5′- AATCGCACACTGAAACGCAC-3′	310 bp	58°C

### Immunoblotting Analysis of Signaling Pathways Activation in Human Fetal Frontal Bone and Parietal Bone Derived Osteoblasts

hFOb and hPOb were collected at different time points of the osteogenic differentiation assay as indicated. Cells were lysate with cold RIPA buffer (50 mmol/L of HEPES, pH 7.5, 150 mmol/L of NaCl, 1 mmol/of EDTA, 10% glycerol, 1% Triton-X-100, 25 mM sodium fluoride) containing 1 mM sodium orthovanadate and Proteases Inhibitor Cocktail (Sigma-Aldrich, St. Louis, MO) followed by three times sonication for 1 min at 3 Watts and a brief spin at 10,000 rpm at 4°C. Total cellular protein was quantified by bicinchoninic acid protein assay (Pierce, Thermo-Fisher Scientific, Waltham, MA). Samples (40–70 μg) were analyzed by electrophoresis on Nu-PAGE 4–12% bis-Tris-HCl sodium dodecyl sulfate-polyacrylamide gel (Novex, Life Technologies, Carlsbad, CA) and transferred onto Immobilon-P membrane (Millipore Corporation, Bedford, MA). Membranes were probed using the following primary rabbit antibodies: anti-phospho-ERK p44/42 (Thr^202^/Tyr^204^) (1:1000; Cell Signaling Technology, Beverly, MA), anti-ERK2 (C-14) (1:400; Santa Cruz Biotechnology, Santa Cruz, CA); anti-phosphorylated SMAD-2 (Ser465/467), anti-SMAD-2, anti-phosphorylated SMAD-1/5 (Ser465/467), anti-SMAD-5 (1:1,000; Cell Signaling Danvers, MA); mouse anti-active-β-catenin (anti-ABC), clone 8E7 (1 mg/ml; Millipore, Tamecula, CA) which specifically detects the active form of β-catenin dephosphorylated on Ser37 or Thr41 was, and anti-β-ACTIN (ab8227) (1:4,000; Abcam, Cambridge, MA). A horseradish peroxidase-conjugated secondary anti-rabbit antibody or ant-mouse was used (1:2,000; Cell Signaling Danvers, MA). Immunoblotted products were visualized by enhanced chemiluminescence (Amersham Biosciences, Buckinghamshire, United Kingdom) according to the manufacturer’s instruction. Membranes were re-probed with anti-ERK anti-SMAD-2, anti-SMAD-1/5 antibodies to rule out that differences in phosphoproteins were outcomes of differential levels of endogenous phosphorylated (pan proteins) and anti-β-ACTIN antibody were used as loading and transfer control for each sample. Densitometry analysis of electrophoretic bands was performed using the ImageJ software program (NIH, Bethesda, MA). The results are the mean ± SD of two independent experiments.

### Cell Seeding on Scaffolds

hFOb and hPOb were trypsinized, washed with PBS, and counted. Cells (250,000) were suspended in 100 μl FCS and seeded on apatite-coated poly (lactic-*co*-glycolic acid) (PLGA) scaffolds (Quarto et al., 2015). To mimic a potential clinical translational context, scaffolds were implanted in calvarial defects 2 h after *in vitro* seeding.

### Animal Surgery

All animal experiments were performed in accordance with Stanford University Animal Care and Use Committee guidelines. To evaluate the *in vivo* healing capacity of hFOb and hPOb on frontal bone and parietal bone defects were created in 9-weeks-old male nude CD1-mice (Charles River Laboratories, Wilmington, MA) using a procedure as previously described (Aalami et al., 2004; Quarto et al., 2010; Senarath-Yapa et al., 2016). Briefly, after anesthesia with an intraperitoneal injection of ketamine 100 mg/kg + xylazine 20 mg/kg + acepromazine 3 mg/kg and disinfection of the surgical site of the mice, non-healing critical 4-mm calvarial defects were created with a trephine drill in parietal bone both, the left and right side carefully avoiding to damage the underlying dura mater or neighboring cranial sutures. Treatment groups included no treatment (empty), scaffold with serum, scaffold seeded either with hFOb or hPOb.

### Micro-CT-Scanning

μCT-scanning was performed as previously described (Aalami et al., 2004; Quarto et al., 2010; Senarath-Yapa et al., 2016) using a high-resolution MicroCAT II scanner (ImTek, Inc., Knoxville, TN) with an x-ray voltage of 80 kVP and an anode current of 450 μA. A resolution of 80 μm was obtained with 144 steps over 360Åã rotation. X-ray data reconstruction was performed with Cobra EXXIM (EXXIM Computing, Corp., Livermore, CA), and Micro View Software (GE Healthcare, Buckinghamshire, United Kingdom). Each mouse was scanned with a CT-phantom to calibrate each scan. The precise threshold of calvarial bone regenerating was previously determined equivalent to 510 Houndsfield Units (Quarto et al., 2010; Senarath-Yapa et al., 2016). The rest-defect area was defined with the Magic Wand Tool in Photoshop (Adobe Systems, San Jose, CA). Percentage healing was determined by dividing the rest-defect area by the mean of the defect size 1 day postoperatively. μCT-scanning was performed in mice 24 h post-surgery and at the indicated time. For statistical analysis, empty group and scaffold alone group was compared with other groups using the Mann-Whitney *U*-test. A ^∗^*p*-value < 0.05 was considered statistically significant.

### Histology

Skulls were harvested under a stereomicroscope and fixed in 10% neutral buffered formalin overnight at 4°C followed by decalcification in 19% EDTA for the appropriate time. Specimens were then dehydrated and paraffin embedded. Movat’s Pentachrome staining was performed on 8 μm coronal sections according to standard procedures. Pentachrome stained sections were examined with Leica DMI 4000B microscope (Leica Biosystems Inc., Buffalo Grove, IL). Images were captured by camera and combined by Adobe Photoshop (Adobe Systems, San Jose, CA).

### Immunohistochemistry for Human Nuclear Antigen

IHC was performed to evaluate the presence of transplanted hFOb and hPOb into mouse calvarial defects 12-weeks post transplantation. Briefly, selected paraffin sections were heated at 50°C for 1 h and allowed to equilibrate at room temperature for 5 min followed by 20 min deparaffinization in xylene and graded ethanol rehydration. Antigen retrieval was performed using a Trypsin Enzymatic Antigen Retrieval Kit (AB970, Abcam, Cambridge, MA) according to the manufacture protocol. Sections were stained using Human Nuclear Antigen Antibody (dilution 1:20, Clone 235-1, MAB1281, Millipore Sigma, Bedford, MA) and M.O.M (Mouse on Mouse) Immunodetection Kit (BMK-2202, Vector Laboratories, Burlingame, CA) with additional overnight blocking and streptavidin/biotin blocking using the Streptavidin/Biotin Blocking Kit (SP-2002, Vector Laboratories, Burlingame, CA) according to manufacture protocols. VECTASTAIN Elite ABC-HRP Kit (PK-6100, Vector Laboratories, Burlingame, CA) was used prior to visualization and development with the DAB Substrate Kit (550880, BD Biosciences, San Jose, CA) following the manufactures protocol. Images are representative of at least 3 independent samples or experiments.

### Statistical Analysis

Data are presented as mean ± SD of three independent experiments. For statistical analysis, empty group and scaffold alone group was compared with other groups using the Mann-Whitney *U*-test. A ^∗^*p*-value < 0.05 was considered statistically significant.

## Data Availability Statement

The raw data supporting the conclusions of this article will be made available by the authors, without undue reservation.

## Ethics Statement

The studies involving human participants were reviewed and approved by the Stanford Institutional Review Board guidelines (IRB-35711). Written informed consent to participate in this study was provided by the participants’ legal guardian/next of kin. The animal study was reviewed and approved by Stanford Institutional Review Board guidelines (IRB-35711).

## Author Contributions

NQ designed and performed the experiments, analyzed the data, and wrote the manuscript. SM performed the experiments and helped with the manuscript writing. JH performed the experiments, helped with the figures preparation, and the manuscript writing. CD performed the experiments and analyzed the data. ML edited the manuscript. All authors contributed to the article and approved the submitted version.

## Conflict of Interest

The authors declare that the research was conducted in the absence of any commercial or financial relationships that could be construed as a potential conflict of interest.

## Publisher’s Note

All claims expressed in this article are solely those of the authors and do not necessarily represent those of their affiliated organizations, or those of the publisher, the editors and the reviewers. Any product that may be evaluated in this article, or claim that may be made by its manufacturer, is not guaranteed or endorsed by the publisher.

## References

[B1] Aalami O. O. Nacamuli R. P. Lenton K. A. Cowan C. M. Fang T. D. Fong K. D. (2004). Applications of a mouse model of calvarial healing: differences in regenerative abilities of juveniles and adults. *Plast. Reconstr. surg.* 114 713–720. 10.1097/01.PRS.0000131016.12754.30 15318051

[B2] Caputo Barucchi V. Giovannotti M. Nisi Cerioni P. Splendiani A. (2013). Genome duplication in early vertebrates: insights from agnathan cytogenetics. *Cytogenet. Genome Res.* 141 80–89. 10.1159/000354098 23949002

[B3] Chaudhary L. R. Avioli L. V. (1997). Activation of extracellular signal-regulated kinases 1 and 2 (ERK1 and ERK2) by FGF-2 and PDGF-BB in normal human osteoblastic and bone marrow stromal cells: differences in mobility and in-gel renaturation of ERK1 in human, rat, and mouse osteoblastic cells. *Biochem. Biophys. Res. Commun.* 238 134–139. 10.1006/bbrc.1997.7256 9299466

[B4] Chen D. Zhao M. Mundy G. R. (2004). Bone morphogenetic proteins. *Growth Factors* 22 233–241. 10.1080/08977190412331279890 15621726

[B5] Clevers H. Loh K. M. Nusse R. (2014). Stem cell signaling. An integral program for tissue renewal and regeneration: wnt signaling and stem cell control. *Science* 346:1248012. 10.1126/science.1248012 25278615

[B6] Davis L. A. Zur Nieden N. I. (2008). Mesodermal fate decisions of a stem cell: the Wnt switch. *Cell. Mol. Life Sci.* 65 2658–2674. 10.1007/s00018-008-8042-1 18528633 PMC2778684

[B7] Fabbri M. Nicolás M. K. Pritchard A. C. Michael H. Eva H. Bever G. S. (2017). The skull roof tracks the brain during the evolution and development of reptiles including birds. *Nat. Ecol. Evol.* 1 1543–1550. 10.1038/s41559-017-0288-2 29185519

[B8] Glaeser J. D. Behrens P. Stefanovic T. Salehi K. Papalamprou A. Tawackoli W. (2021). Neural crest-derived mesenchymal progenitor cells enhance cranial allograft integration. *Stem Cells Transl. Med.* 10 797–809. 10.1002/sctm.20-0364 33512772 PMC8046069

[B9] Hartmann C. A. (2006). Wnt canon orchestrating osteoblastogenesis. *Trends Cell Biol.* 16 151–158. 10.1016/j.tcb.2006.01.001 16466918

[B10] Homayounfar N. Park S. S. Afsharinejad Z. Bammler T. K. MacDonald J. W. Farin F. M. (2015). Transcriptional analysis of human cranial compartments with different embryonic origins. *Arch. Oral Biol.* 60 1450–1460. 10.1016/j.archoralbio.2015.06.008 26188427 PMC4750879

[B11] Jiang X. Iseki S. Maxson R. E. Sucov H. M. Morriss-Kay G. M. (2002). Tissue origins and interactions in the mammalian skull vault. *Dev. Biol.* 241 106–116. 10.1006/dbio.2001.0487 11784098

[B12] Kanzler B. Foreman R. K. Labosky P. A. Mallo M. (2000). BMP signaling is essential for development of skeletogenic and neurogenic cranial neural crest. *Development* 127 1095–1104. 10.1242/dev.127.5.1095 10662648

[B13] Krishnan V. Bryant H. U. Macdougald O. A. (2006). Regulation of bone mass by Wnt signaling. *J. Clin. Investig.* 116 1202–1209. 10.1172/JCI28551 16670761 PMC1451219

[B14] Le Lièvre C. S. Le Douarin N. M. (1975). Mesenchymal derivatives of the neural crest: analysis of chimaeric quail and chick embryos. *J. Embryol. Exp. Morphol.* 34 125–154. 10.1242/dev.34.1.125 1185098

[B15] Li S. Meyer N. P. Quarto N. Longaker M. T. (2013). Integration of multiple signaling regulates through apoptosis the differential osteogenic potential of neural crest-derived and mesoderm-derived Osteoblasts. *PLoS One* 8:e58610. 10.1371/journal.pone.0058610 23536803 PMC3607600

[B16] Li S. Quarto N. Longaker M. T. (2010). Activation of FGF signaling mediates proliferative and osteogenic differences between neural crest derived frontal and mesoderm parietal derived bone. *PLoS One* 5:e14033. 10.1371/journal.pone.0014033 21124973 PMC2987799

[B17] Logan C. Y. Nusse R. (2004). The Wnt signaling pathway in development and disease. *Annu. Rev. Cell Dev. Biol.* 20 781–810. 10.1146/annurev.cellbio.20.010403.113126 15473860

[B18] Monroe D. G. McGee-Lawrence M. E. Oursler M. J. Westendorf J. J. (2012). Update on Wnt signaling in bone cell biology and bone disease. *Gene* 492 1–18. 10.1016/j.gene.2011.10.044 22079544 PMC3392173

[B19] Morriss-Kay G. M. (2001). Derivation of the mammalian skull vault. *J. Anat.* 199 143–151. 10.1046/j.1469-7580.2001.19910143.x 11523816 PMC1594961

[B20] Noël D. Gazit D. Bouquet C. Apparailly F. Bony C. Plence P. (2004). Short-term BMP-2 expression is sufficient for in vivo osteochondral differentiation of mesenchymal stem cells. *Stem Cells* 22 74–85. 10.1634/stemcells.22-1-74 14688393

[B21] Nusse R. (2005). Wnt signaling in disease and in development. *Cell Res.* 15 28–32. 10.1038/sj.cr.7290260 15686623

[B22] Nusse R. (2008). Wnt signaling and stem cell control. *Cell Res.* 18 523–527. 10.1038/cr.2008.47 18392048

[B23] Opperman L. A. (2000). Cranial sutures as intramembranous bone growth sites. *Dev. Dyn.* 219 472–485. 10.1002/1097-0177(2000)9999:9999<::AID-DVDY1073>3.0.CO;2-F 11084647

[B24] Quarto N. Longaker M. T. (2005). The zebrafish (Danio rerio): a model system for cranial suture patterning. *Cells Tissues Organs* 181 109–118. 10.1159/000091100 16534205

[B25] Quarto N. Behr B. Li S. Longaker M. T. (2009). Differential FGF ligands and FGF receptors expression pattern in frontal and parietal calvarial bones. *Cells Tissues Organs* 190 158–169. 10.1159/000202789 19218784 PMC2820336

[B26] Quarto N. Leonard B. Li S. Marchand M. Anderson E. Behr B. (2012). Skeletogenic phenotype of human Marfan embryonic stem cells faithfully phenocopied by patient-specific induced-pluripotent stem cells. *Proc. Natl. Acad. Sci. U. S. A.* 109 215–220. 10.1073/pnas.1113442109 22178754 PMC3252902

[B27] Quarto N. Senarath-Yapa K. Renda A. Longaker M. T. (2015). TWIST1 silencing enhances *in vitro* and *in vivo* osteogenic differentiation of human adipose-derived stem cells by triggering activation of BMP-ERK/FGF signaling and TAZ upregulation. *Stem Cells* 33 833–847. 10.1002/stem.1907 25446627 PMC5720150

[B28] Quarto N. Wan D. C. Kwan M. D. Panetta N. J. Li S. Longaker M. T. (2010). Origin matters: differences in embryonic tissue origin and Wnt signaling determine the osteogenic potential and healing capacity of frontal and parietal calvarial bones. *J. Bone Miner. Res.* 25 1680–1694. 10.1359/jbmr.091116 19929441 PMC3154006

[B29] Senarath-Yapa K. Li S. Walmsley G. G. Zielins E. Paik K. Britto J. A. (2016). Small Molecule Inhibition of Transforming Growth Factor Beta Signaling Enables the Endogenous Regenerative Potential of the Mammalian Calvarium. *Tissue Eng. Part A* 22 707–720. 10.1089/ten.tea.2015.0527 27036931 PMC4876548

[B30] Srinivasan A. Teo N. Poon K. J. Tiwari P. Ravichandran A. Wen F. (2021). Comparative Craniofacial Bone Regeneration Capacities of Mesenchymal Stem Cells Derived from Human Neural Crest Stem Cells and Bone Marrow. *ACS Biomater. Sci. Eng.* 7 207–221. 10.1021/acsbiomaterials.0c00878 33455206

[B31] Yoshida T. Vivatbutsiri P. Morriss-Kay G. Saga Y. Iseki S. (2008). Cell lineage in mammalian craniofacial mesenchyme. *Mech. Dev.* 125 797–808. 10.1016/j.mod.2008.06.007 18617001

